# *Dodonaea viscosa* Jacq. induces cytotoxicity, antiproliferative activity, and cell death in colorectal cancer cells via regulation of caspase 3 and p53

**DOI:** 10.3389/fphar.2023.1197569

**Published:** 2023-06-23

**Authors:** Oscar Herrera-Calderon, Angie Herrera-Ramírez, Wilson Cardona-G, Elizabeth Julia Melgar-Merino, Haydee Chávez, Josefa Bertha Pari-Olarte, Eddie Loyola-Gonzales, José Francisco Kong-Chirinos, José Santiago Almeida-Galindo, Gilmar Peña-Rojas, Vidalina Andía-Ayme

**Affiliations:** ^1^ Department of Pharmacology, Bromatology, and Toxicology, Faculty of Pharmacy and Biochemistry, Universidad Nacional Mayor de San Marcos, Lima, Peru; ^2^ Chemistry of Colombian Plants Group, Institute of Chemistry, Faculty of Exact and Natural Sciences, University of Antioquia (UdeA), Medellín, Colombia; ^3^ Department of Chemistry Sciences, Faculty of Pharmacy and Biochemistry, Universidad Nacional San Luis Gonzaga, Ica, Peru; ^4^ Department of Pharmaceutical Chemistry, Faculty of Pharmacy and Biochemistry, Universidad Nacional San Luis Gonzaga, Ica, Peru; ^5^ Department of Pharmaceutical Sciences, Faculty of Pharmacy and Biochemistry, Universidad Nacional San Luis Gonzaga, Ica, Peru; ^6^ Department of Surgical Clinical Sciences, Faculty of Human Medicine, Universidad Nacional San Luis Gonzaga, Ica, Peru; ^7^ Department of Basic Sciences, Faculty of Human Medicine, Universidad Nacional San Luis Gonzaga, Ica, Peru; ^8^ Laboratory of Cellular and Molecular Biology, Biological Sciences Faculty, Universidad Nacional de San Cristóbal de Huamanga, Ayacucho, Peru; ^9^ Food Microbiology Laboratory, Biological Sciences Faculty, Universidad Nacional de San Cristóbal de Huamanga, Ayacucho, Peru

**Keywords:** *Dodonaea viscosa*, medicinal plant, colorectal cancer, phytochemical screening, cell death

## Abstract

Colorectal cancer (CRC) is the third most common cancer diagnosed worldwide and is the second leading cause of cancer-related death due to an insufficiency prognosis and is generally diagnosed in the last step of development. The Peruvian flora has a wide variety of medicinal plants with therapeutic potential in several diseases. *Dodonaea viscosa* Jacq. is a plant used to treat inflammatory process as well as gastrointestinal diseases. The aim of this study was to examine the cytotoxic, antiproliferative, and cell death-inducing effects of *D. viscosa* on colorectal cancer cells (SW480 and SW620). The hydroethanolic extract was obtained by maceration at 70% ethanol, the phytochemical constituents were identified by LC-ESI-MS. *D. viscosa* revealed 57 compounds some of them are: isorhamnetin, kaempferol, quercetin, methyl dodovisate B, hardwickiic acid, viscosol, and dodonic acid. Regarding the antitumoral activity, *D. viscosa* induced cytotoxic and antiproliferative activity in both SW480 and SW620 cancer cells, accompanied with, important changes in mitochondrial membrane potential, formation of the Sub G0/G1 population and increasing levels of apoptotic biomarkers (caspase 3 and the tumor suppressor protein p53) in the metastatic derivative cell line (SW620), suggesting an intrinsic apoptotic process after the treatment with the hydroethanolic extract of *D. viscosa*.

## 1 Introduction

According to GLOBOCAN 2020, colorectal cancer (CRC) is one of the most prevalent gastrointestinal cancers diagnosed globally, ranking third in men and second in women (Pitchumoni and Broder, 2020; Pitchumoni and Broder, 2020; Ferlay et al., 2021; Ferlay et al., 2021). In addition, China and the United States are projected to have the largest number of new cases of CRC over the next 2 decades, with an estimated 3,2 million new cases worldwide by 2040 (Xi and Xu, 2021). CRC is the fourth most prevalent cancer in Latin America, with an incidence of 16.6 cases per 100,000 inhabitants, and Uruguay with the highest incidence rate (GCO, 2022). In Peru, CRC ranks fourth for both sexes, third for men and fourth for women (Ferlay et al., 2021).

Regarding CRC, several molecular processes are implicated in the formation of CRC, including mutational inactivation of tumor-suppressor genes p53, adenomatous polyposis coli (APC), transforming growth factor-α (TGF-α), and activation of oncogene pathways (PI3K, RAS, and BRAF). In recent years, phytoextracts and phytoconstituents derived from plants have gained growing attention for their possible anti-CRC properties (Liu et al., 2022). The antiproliferative, cytotoxic, antimutagenic, and anticancer phytochemical substances are abundant in medicinal plants (Martínez-Aledo et al., 2020) and might be acting by regulating p53 levels, these extracts have been shown to decrease proliferation and tumor angiogenesis, induce apoptosis, and block the proliferation of tumor cells at several phases, including G2/M, G1/S, S phase, G0/G1, and G1 phase (Aiello et al., 2019).

*Dodonaea viscosa* Jacq. (Sapindaceae family) has been studied as a potential plant for chronic disorders in Peruvian traditional medicine (Herrera-Calderon et al., 2020). The leaves of this plant, known in Spanish as “shamana” or “chamana,” have shown anti-inflammatory and analgesic properties (Herrera-Calderon et al., 2020; Herrera-Calderon et al., 2022). Additionally, the anticancer properties of *D. viscosa* were evaluated against a human lung adenocarcinoma cell line (A549 NSCLC cell) using a cytotoxic extract of the plant’s leaves (Anandan and Gurumallesh Prabu, 2018). Another study confirmed the cytotoxic effect of the leaf extract against colon cancer cells (HT-29) (Herrera-Calderon et al., 2020). In addition, considering that one of the possible mechanisms to fight against cancer cells could be mediated by radical scavenging, a recent investigation showed that *D. viscosa* could exert a potent antioxidant effect, due to the wide variety of polyphenols detected in various solvent extracts of this plant. Furthermore, the ethanol extract of stem plus ethyl acetate extract of the root had antiproliferative activity against THP-1 (human leukemia monocytic cells) and Hep G2 cell lines (hepatocellular carcinoma) (Malik et al., 2022). Other cell lines treated with *D. viscosa* included carcinoma cell lines from human lung cancer (A549) and ovarian cancer (SK-OV-3) (Al-Musawi and Al-Saadi, 2021) All these properties make this plant an attractive source of study for discovering of new therapeutic alternatives against colorectal cancer.

This study focuses on the phytochemical analysis of the hydroethanolic extract of *D. viscosa* and the determination of its anticancer effect on colorectal cancer cell lines *in vitro* (SW480 and SW620). The antitumor effect includes several steps to understand how the extract works on these tumor cell lines, including cytotoxicity, antiproliferative, changes in mitochondrial membrane potential (ΔΨm) and plasma membrane integrity, its effect on cell cycle distribution, induction of apoptosis, and determination of apoptotic biomarkers such as caspase 3 and the tumor suppressor protein p53.

## 2 Materials and methods

### 2.1 Plant material

Leaves of *D. viscosa* were collected in Chalhuanca, province of Aymaraes, in the department of Apurimac, Peru (2,880 masl) in July 2019. This plant was authenticated by Prof. Asunción Cano at the Herbarium of the Natural History Museum, Universidad Nacional Mayor de San Marcos, with a voucher specimen (125-USM-2019).

### 2.2 Preparation of the hydroethanolic extract

The powdered leaves (500 g) were extracted with 70% ethanol at 25°C for 7 days. Afterward, the hydroethanolic extract was filtered using a Buchner apparatus, and the filtrates was incorporated into a rota-evaporator to remove the solvent, resulting in a dark green solid extract. The hydroalcoholic extract weighed 34.02 g with a yield percentage of 6.8%

### 2.3 UPLC-ESI-MS/MS

#### 2.3.1 Sample preparation

A quantity of 20 mg of the hydroethanol extract was extracted using an ultrasonicator (40 kHz, heat power 150 W; Branson 3,800, MO, United States) with 100 mL of MeOH-H_2_O (8:2) for 30 min. The solution was filtered into a vial and then 1.0 mL of the solution was injected into the LC-MS system.

#### 2.3.2 UPLC-ESI-MS/MS analysis of the hydroethanolic extract of *D. viscosa*

The sample was injected on a Dionex Ultimate 3000 UHPLC Systems (Thermo Scientific) triple triple-quadrupole instrument and a mass spectrometer, Q Exactive Plus (Thermo Scientific) with a column Luna^©^ Omega C18 100 Å, Phenomenex (150 mm × 2.1 mm, 1.6 μm), at 0.3 min/mL flow rate and a column temperature 30°C. The mobile phase consisted of two phases; A: 1% formic acid, B: acidified acetonitrile containing 1% formic acid. The gradient conditions were 0–1 min, B 10%; 1–20 min, B 10%–95%; 20–21 min, B 95%; 21–23 min, B 95%–10%; and 23–30 min, B 10%. The ionization source parameters were set using a positive and negative ion mode as follows: spray voltage 3.5/2.5 KV; capillary temperature 260°C; gas carrier N2 (sheath gas flow rate 48, sweep gas flow rate 1); gas heater temperature 300°C; S-lens RF level 100; normalized collision energy 30. Full MS scan parameters: range 120–1,500 m/z; resolution 35,000; microscans 1; AGC target 5 × 10^6^; maximum IT 80 ms. MS^2^ parameters: resolution 17,500; AGC target 1 × 10^6^; maximum IT 100 ms (Herrera-Calderón et al., 2021).

Data acquisition and processing were performed with a Thermo XcaliburTM software version 3.0 (Thermo Fisher Scientific Inc., Waltham, MA, United States) with the Qual Browser, and metabolite annotations were performed with MS-Dial software version 4.70 (Riken, Osaka University, Suita City, Japan) using the MS-Dial metabolomics MPS spectral kit library (available at: https://prime.psc.riken.jp/compms/msdial/main.html; last updated on 13 April 2021).

### 2.4 *In vitro* biological assays

#### 2.4.1 Cell lines and culture medium

Two different human colorectal cancer cell lines (SW480 and SW620) and noncancerous cells (human keratinocytes, HaCaT; Chinese hamster ovary, CHO-K1) were used in this study. These were purchased from European Collection of Authenticated Cell Cultures (ECACC, England). Cell cultures were maintained at 37°C in Dulbecco’s Modified Eagle Medium. Culture medium was supplemented with 1% non-essential amino acids (Gibco Invitrogen, Carlsbad, United States), 1% penicillamine/streptomycin, and 10% heat-inactivated horse serum. For all the experiments, the horse serum in the growing medium was reduced to 3% and it was supplemented with insulin (10 mg/mL), transferrin (5 mg/mL) and selenium (5 ng/mL) (ITS-defined medium, Gibco, *Invitrogen*, Carlsbad, United States) (Herrera et al., 2018).

#### 2.4.2 Cytotoxic activity of the hydroethanolic extract of *D. viscosa*

The cytotoxicity of *D. viscosa* and the reference material were evaluated *in vitro* using Sulforhodamine B (SRB), a colorimetric approach that can detect cellular protein of live cells. SW480, SW620, and CHO-k1 cells were seeded at a density of 20,000 cells per well, whereas HaCaT cells were seeded at a density of 10,000 cells per well on 96-well tissue culture plates. The cells were then incubated at 37°Cinto a humidified environment containing 5% CO_2_. After 24 h of cell adhesion, cell cultures were treated with the vehicle control (1% DMSO) or different extract doses (5–320 μg/mL). The cell lines were then fixed for 1 h at 4°C using trichloroacetic acid (50% v/v) (Merck, Bogotá, Colombia). Cell cultures were stained with SRB (Sigma-Aldrich, United States) for 30 min at room temperature before being washed with 1% acetic acid. Tris-base was utilized to solubilize protein-bound SRB to quantify absorbance at 492 nm with a microplate reader (Mindray MR-96A) (10 mM). A minimum of three repetitions were done for each experiment (Herrera et al., 2021). The selectivity index (SI) was calculated to determine the cytotoxic selectivity of the evaluated substances based on the following formula: IC_50_ of the normal cells (HaCaT and CHO-K1)/IC_50_ of the tumor cells (SW480 and SW620). If SI is more than 1, the substance was more cytotoxic to tumor cells than normal cells.

#### 2.4.3 Antiproliferative activity of the hydroethanolic extract of *D. viscosa*

The antiproliferative properties of *D. viscosa* were evaluated using the same previously describe technique of SRB with minor modifications. Briefly, 2,500 cells (SW480 and SW620) were seeded in 96-well tissue culture plates. After 24 h of cell adhesion, cell cultures were exposed to increasing concentrations of the extract (5–160 μg/mL; concentrations based on IC_*50*_ values) or DMSO (vehicle control, 1%), from day 0 to day 8. The culture medium with the hydroethanolic extract was replaced every 48 h. Cells were fixed, stained, and read as previously described in the section of cytotoxic activity (2.4.2), using trichloroacetic acid (50% v/v), SRB and washing with acetic acid to eliminate the excess dye. The reading process was carried out at 492 nm (Herrera et al., 2018).

#### 2.4.4 Double staining for mitochondrial membrane potential (ΔΨm) and plasma membrane integrity

After 48 h of treatment with the *D. viscosa* hydroethanolic extract, the mitochondrial membrane potential was calculated using double fluorescence staining with propidium iodide (PI) and DiOC6(3). Cells were scrapped using the same culture medium in which they were seeded. After that, the media was removed through centrifugation. The pellet was resuspended in 500 μL of versine buffer containing DiOC6(3) and PI from Thermo Fisher Scientific in Waltham, Massachusetts, United States. At the end, the cell suspension was kept at room temperature for 30 min in complete darkness and 10,000 events were counted using flow cytometry (Herrera et al., 2019).

#### 2.4.5 Effect of the hydroethanolic extract of *D. viscosa* on cell cycle distribution

Flow cytometry was used to assess the results of the cell cycle analyses using propidium iodide (PI),. Following a time of 48-h of treatment using DMSO (1%) as a vehicle control and the IC_50_ value of the *D. viscosa* extract. Cells were further collected by scraping and centrifugation, resuspending the cell pellet in versene buffer. Then, the fixation process with 1.8 mL 70% ethanol was carried out at 4°C for 1 h. After washing with versene buffer, the alcohol was eliminated. The final pellet was resuspended in 300 µL of PBS with 0.25 mg/mL RNAse (Type I-A, Sigma-Aldrich, Germany) and 0.1 mg/mL PI, and incubated at room temperature for 30 min in the dark. The PI fluorescence of 10,000 events was read using a FACS Canto II flow cytometer (BD Biosciences, United States). The software FlowJo 7.6.2 (Ashland, OR, United States) was used to analyze the data (Herrera et al., 2021).

#### 2.4.6 Cell death induction by the hydroethanolic extract of *D. viscosa*

Using Annexin V/FITC and PI (Roche Diagnostics), in accordance with the manufacturer’s instructions, membrane damage and phosphatidylserine exposure were examined. The *D. viscosa* hydroethanolic extract was applied to SW480 and SW620 cells for 48 h before the cells were scraped from the surface and collected. The cell pellet was centrifuged-washed, resuspended in a solution containing Annexin V/FITC - PI, and incubated for 20 min in complete darkness. Utilizing the software FlowJo 7.6.2, data were collected using a flow cytometry (Ashland, OR, United States). Individual Annexin V/FITC staining was visible in cells that were in the early stages of apoptosis. Cells that stained positively for PI were categorized as being dead, late apoptotic, necroptotic, or secondary necrotic cells. Assays were carried out in duplicate.

#### 2.4.7 Determination of apoptotic biomarkers

After 48 h of exposure to *D. viscosa* hydroethanolic extract, cell lines were scraped and collected using Cell Lysis Buffer (1X, Reference #988). The supernatant was utilized to test the effect of the extract on the modification of several apoptotic markers. Cell-Signaling Technology (Danvers, Massachusetts, United States) supplied the kits for cleaved caspase-3 and p53, whereas Elabscience Biotechnology Company supplied the kits for caspases −7 and −8. (China). These tests were conducted under the manufacturer’s instructions (Herrera et al., 2019).

### 2.5 Statistical analysis

The results are presented as the mean SE (standard error) of at least two separate experiments. Utilizing one-way ANOVA and Dunnett’s *post hoc* test, statistical differences were evaluated. *p* values below 0.05 were considered significant. GraphPad Prism 7.04 for Windows was utilized for data analysis (Graph Pad Software, San Diego, California, United States).

## 3 Results and discussion

### 3.1 Phytochemical profile by UPLC-ESI-MS/MS

**FIGURE 1:**
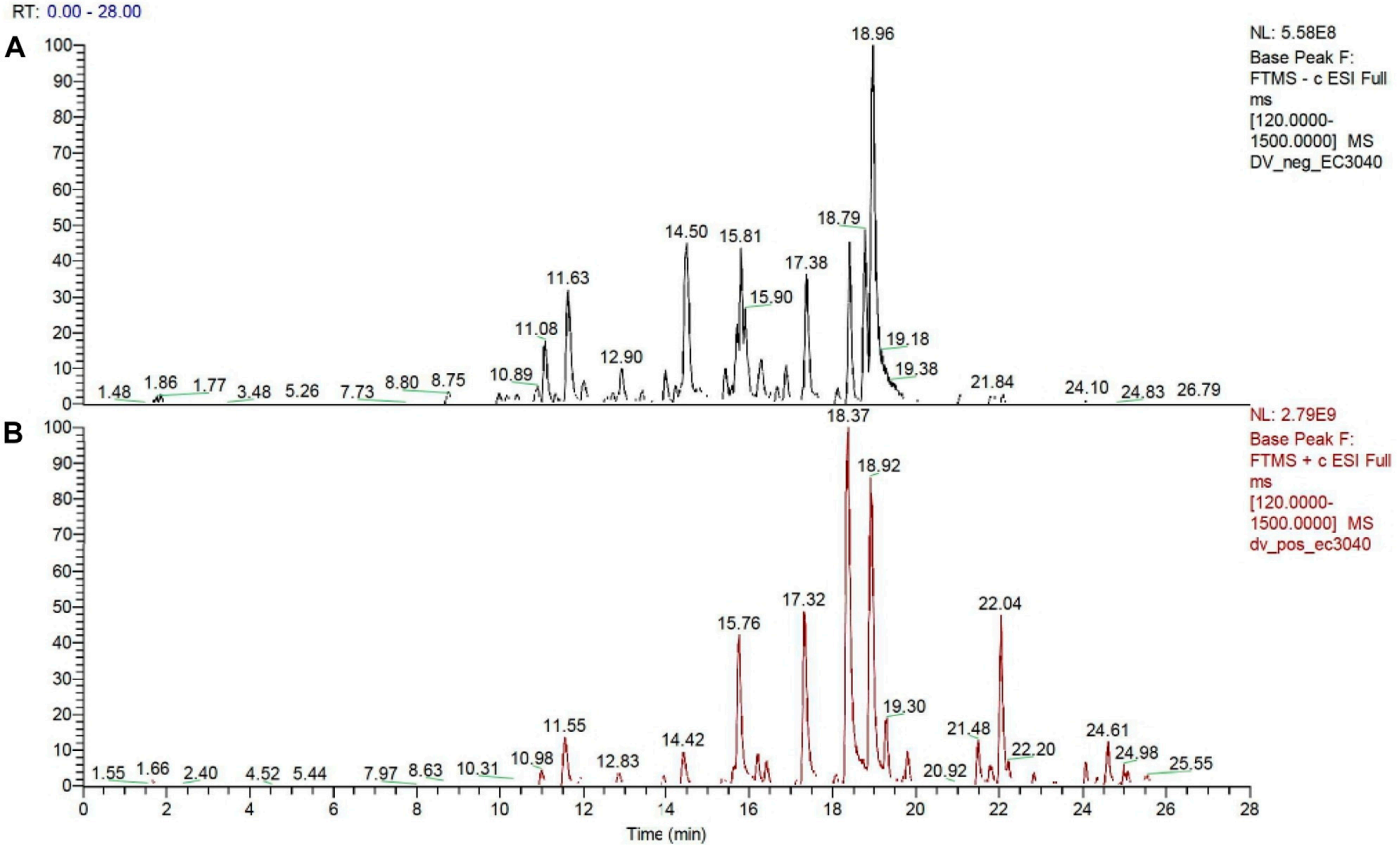
Chromatograms in negative **(A)** and positive **(B)** ESI mode of the hydroethanolic extract of *D. viscosa*.

The maceration of *D. viscosa* leaf powder in ethanol produced a dark green extract with a yield of 6.8% (w/w dry powder). The phytochemical constituents identified by LC-ESI-MS/MS are depicted in a positive and negative modes in Figure 1. Each retention time corresponds to a determined phytochemical constituent. The chromatographic analysis revealed 57 components, primarily phenolic compounds and diterpenes. Nine were detected in ESI (−), thirty-two were observed in ESI (+), and sixteen were observed in both modes (Table 1; Supplementary Table S1). Figure 1 depicts the positive and negative ESI chromatographic profiles for the hydroethanolic extract of *D. viscosa* leaves.

**TABLE 1:** Chemical constituents determined in the hydroethanolic extract of *D. viscosa* Jacq leaves using UPLC-ESI-MS/MS.

N°	Retention time (min)	Theoretical mass (Neutral form)	Molecular formula (Neutral form)	Predicted compound	Chemical group	Reference
1	1.68	342.30	C_12_H_22_O_11_	Sucrose 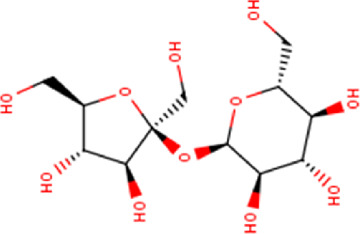	Glycosyl glycoside	—
2	1.79	192.17	C_7_H_12_O_6_	Quinic acid 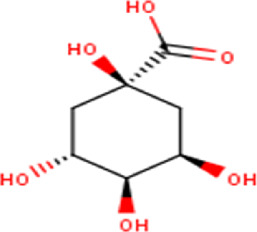	Organic acid	—
3	1.86	174.15	C_7_H_10_O_5_	Shikimic acid 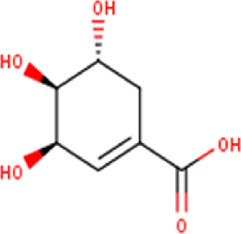	Organic acid	—
4	2.39	267.24	C_10_H_13_N_5_O_4_	Adenosine 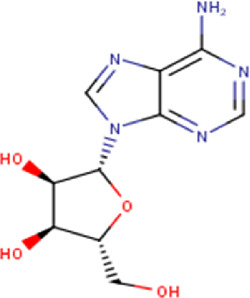	Ribonucleoside	—
5	2.39	293.31	C_12_H_23_NO_7_	N-(1-deoxy-1-fructosyl) isoleucine 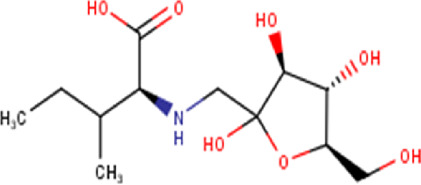	Aminoacid	—
6	5.45	204.22	C_11_H_12_N_2_O_2_	Tryptophan 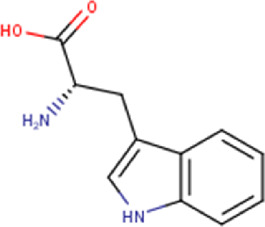	Aminoacid	—
7	7.54	354.31	C_16_H_18_O_9_	6,8-C-dihexosylnoreugenin 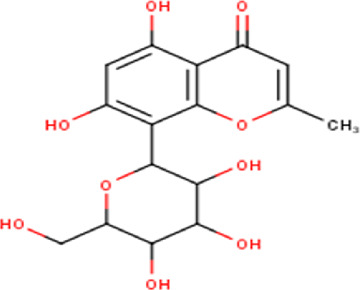	Chromones	—
8	8.75	354.31	C_16_H_18_O_9_	Chlorogenic acid 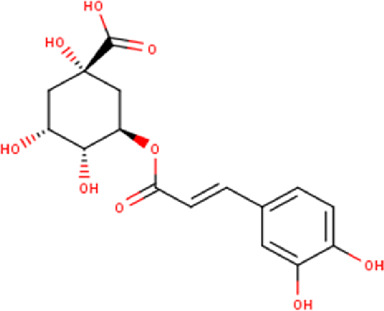	Phenolic acid	Malik et al. (2022)
9	9.03	456.34	C_27_H_32_O_5_	6,8-hexosylnaringenin 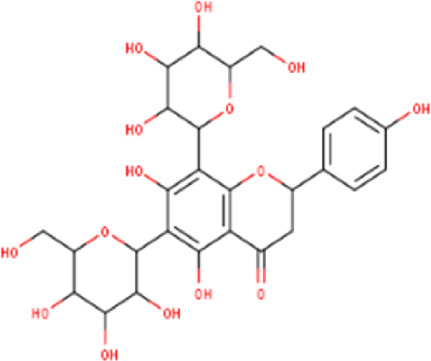	Flavonoid glycosides	—
10	9.52	578.529	C_30_H_26_O_12_	Procyanidin B2 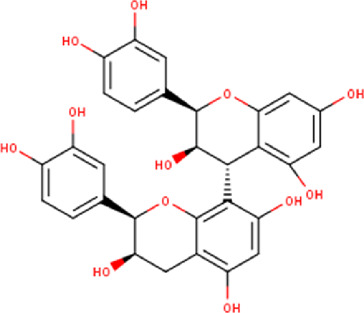	Catechin	—
11	9.95	290.27	C_15_H_14_O_6_	Catechin 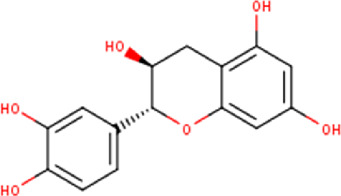	Catechin	Malik et al. (2022)
12	10.23	372.32	C_16_H_20_O_10_	6-(3-benzoyloxy-2-hydroxypropoxy)-3,4,5- trihydroxyoxane-2-carboxylic acid 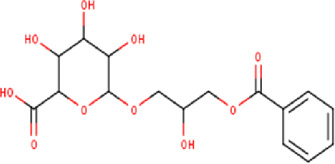	Organic acid	—
13	10.27	338.31	C_16_H_18_O_8_	p-Coumaroylquinic acid 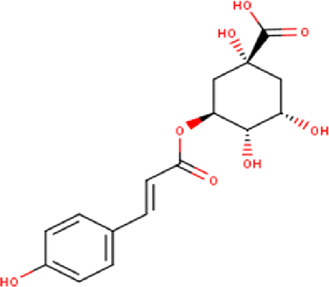	Organic acid	Malik et al. (2022)
14	10.35	756.66	C_33_H_40_O_20_	Quercetin 3-O-[rhamnosyl-(1→2)- [rhamnosyl-(1→6)]-glucoside] 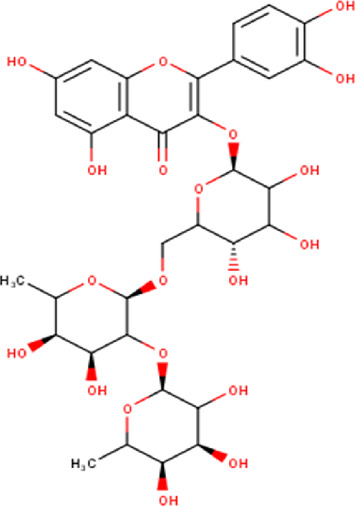	Flavonoid glycosides	—
15	10.40	864.12	C_45_H_36_O_18_	Unknown (Procyanidin trimer)	—	—
16	10.64	368.34	C_17_H_20_O_9_	3-O-Feruloylquinic acid 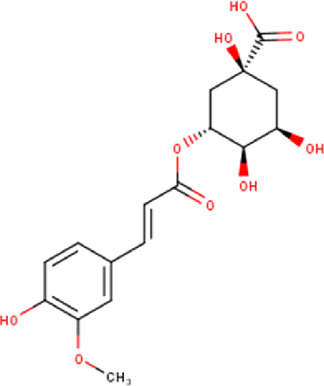	Organic acid	—
17	10.72	740.66	C_33_H_40_O_19_	Kaempferol-3-O-[rhamnosyl-(1→2)- [rhamnosyl-(1→6)]-galactoside] 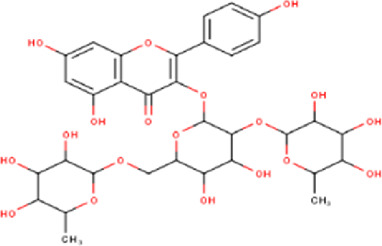	Flavonoid glycosides	—
18	10.85	382.36	C_18_H_22_O_9_	1-O-methyl-2-acetyl-3-p-coumaryl-myo- inositol 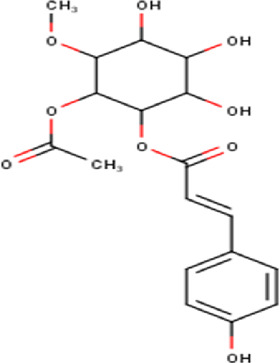	Organic acid	—
19	11.08	610.53	C_27_H_30_O_16_	Rutin 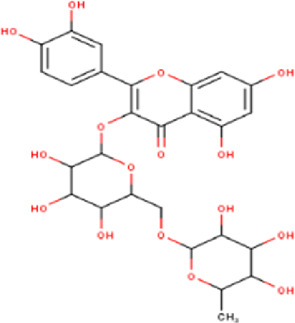	Flavonoid glycosides	Malik et al. (2022)
20	11.42	594.52	C_27_H_30_O_15_	kaempferol-3-O-rutinoside 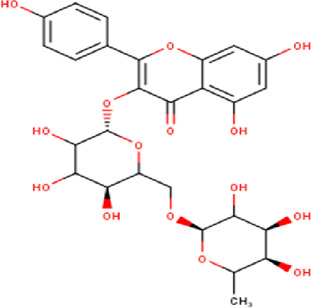	Flavonoid glycosides	—
21	11.63	624.55	C_28_H_32_O_16_	Isorhamnetin 3-O-[α-L-Rhamnopyranosyl- (1→6)-β-D-galactopyranoside 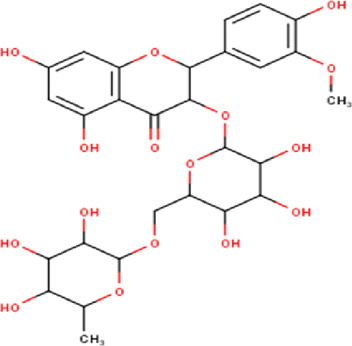	Flavonoid glycosides	—
22	12.21	198.26	C_11_H_18_O_3_	Loliolide 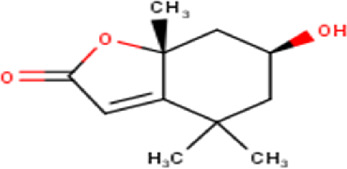	Benzofuran	—
23	12.90	464.52	C_24_H_34_O_9_	Unknown	—	
24	13.11	288.25	C_15_H_12_O_6_	4′,5,7-Trihydroxydihydroflavonol 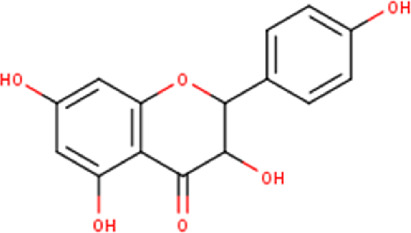	Flavonoid	Sagara et al. (2021)
25	13.40	352.47	C_20_H_32_O_5_	3,8,16-Trihydroxy-13-labden-15,16-olide 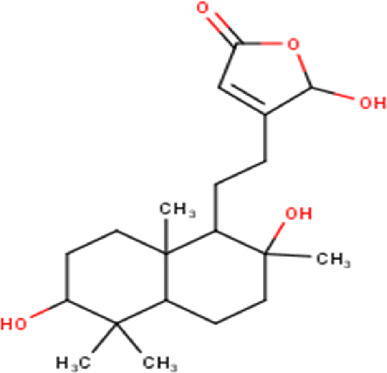	Diterpenoid	—
26	13.45	652.60	C_30_H_36_O_16_	5, 7-dihydroxy-3′,4′,5′-trimethoxyfiavone 7-O-[β-D-glucuronopyranosyl-(1→2)-β-Dglucopyranoside] 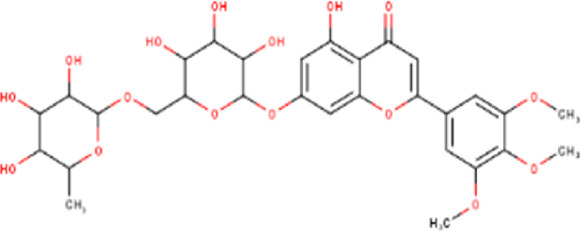	Flavonoid glycosides	—
27	14.00	380.52	C_22_H_36_O_5_	15,16-Epoxy-6,13,14,15,16-pentahydroxy-3- cleroden-18-oic acid, 15,16-dimethyl ether (isomer 1) 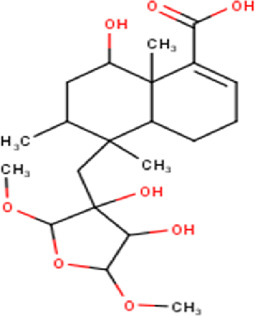	Diterpenoid	Sagara et al. (2021)
28	14.24	380.52	C_22_H_36_O_5_	15,16-Epoxy-6,13,14,15,16-pentahydroxy-3- cleroden-18-oic acid, 15,16-dimethyl ether (isomer 2) 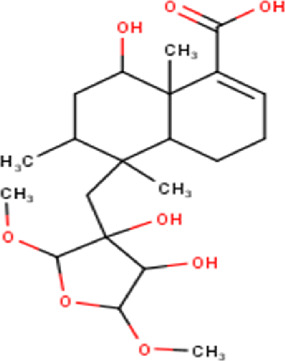	Diterpenoid	Sagara et al. (2021)
29	14.50	364.43	C_20_H_28_O_6_	6,12-dioxo-7-labdene-15,18-dioic acid (isomer 1) 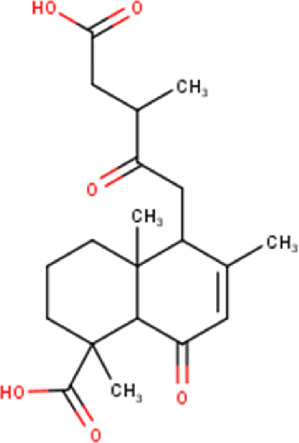	Diterpenoid	Sagara et al. (2021)
30	15.11	272.25	C_15_H_12_O_5_	Naringenin 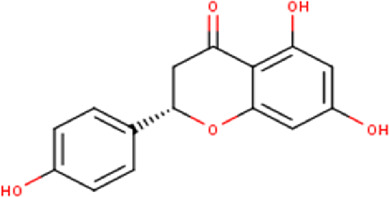	Flavonoid	—
31	15.33	316.44	C_20_H_28_O_3_	Kauralexin B3 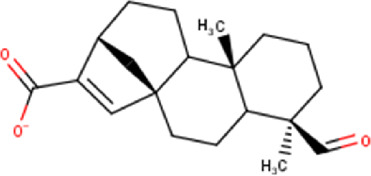	Diterpenoid	—
32	15.45	396.48	C_21_H_32_O_7_	Unknown 1 (isomer 1)	—	—
33	15.71	396.48	C_21_H_32_O_7_	Unknown 1 (Isomer 2)	—	—
34	15.81	330.29	C_17_H_14_O_7_	4′,5,7-Trihydroxy-3,6-dimethoxyflavone 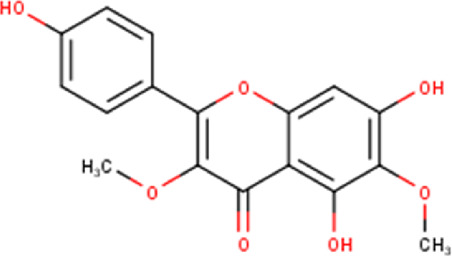	Flavonoid	Zhang et al. (2012)
35	15.81	348.43	C_20_H_28_O_5_	6-Hydroxy-3,13-clerodadien-16,15-olid-18- oic acid 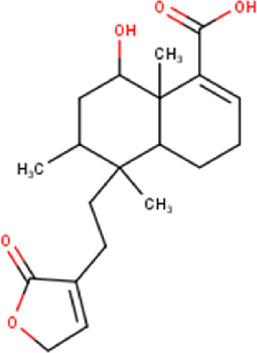	Diterpenoid	Sagara et al. (2021)
36	15.91	364.439	C_20_H_28_O_6_	6,12-dioxo-7-labdene-15,18-dioic acid (isomer 2) 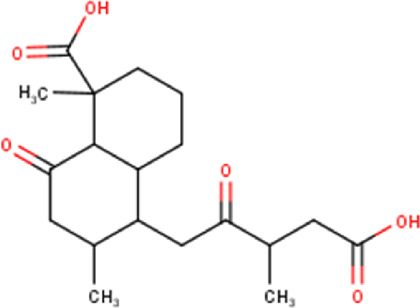	Diterpenoid	Sagara et al. (2021)
37	16.13	302.28	C_16_H_14_O_6_	3′,5,5′-Trihydroxy-7-methoxy flavanone 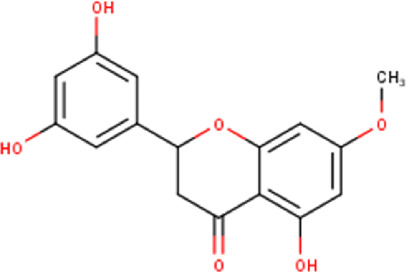	Flavonoid	—
38	16.25	416.42	C_22_H_24_O_8_	Aliarin 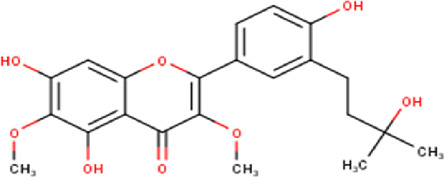	Flavonoid	Sagara et al. (2021)
39	16.28	364.43	C_20_H_28_O_6_	6,12-dioxo-7-labdene-15,18-dioic acid (isomer 3) 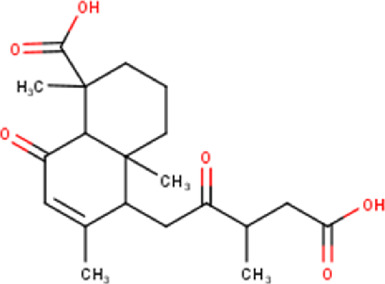	Diterpenoid	Sagara et al. (2021)
40	16.41	316.26	C_16_H_12_O_7_	Tamarixetin 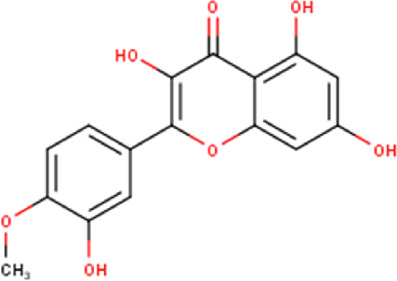	Flavonoid	
41	16.68	346.42	C_20_H_26_O_5_	Unknown 2 (Isomer 1)	—	
42	16.88	346.42	C_20_H_26_O_5_	Unknown 2 (Isomer 2)	—	
43	17.19	344.32	C_18_H_16_O_7_	Penduletin 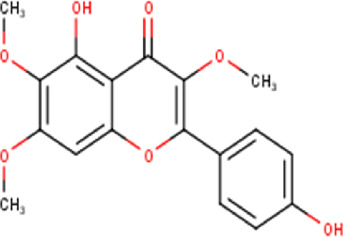	Flavonoid	Muhammad et al. (2015)
44	17.38	286.28	C_16_H_14_O_5_	Sakuratenin 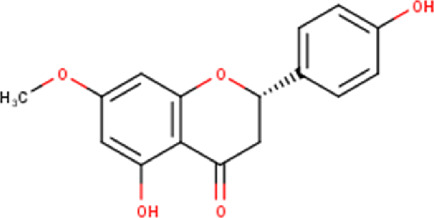	Flavonoid	Zhang et al. (2012)
45	18.11	314.29	C_17_H_14_O_6_	3,5-dihydroxy-4,7-dimethoxy flavone 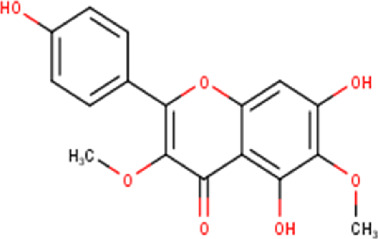	Flavonoid	Muhammad et al. (2015)
46	18.39	344.32	C_18_H_16_O_7_	Santin 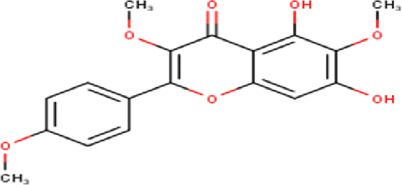	Flavonoid	Muhammad et al. (2015)
47	18.79	330.42	C_20_H_26_O_4_	Mkapwanin 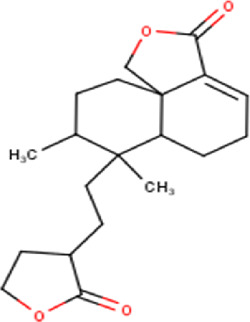	Diterpenoid	—
48	18.96	332.44	C_20_H_28_O_4_	Dodonic acid 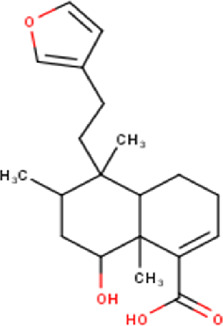	Diterpenoid	Sagara et al. (2021)
49	19.33	398.41	C_22_H_22_O_7_	Dodoviscin J 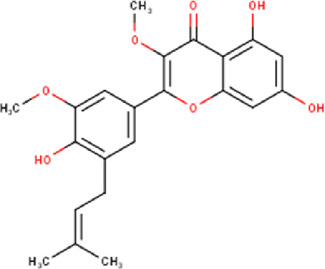	Flavonoid	Gao et al. (2013)
50	19.57	358.47	C_22_H_30_O_4_	Dodovisnoid E 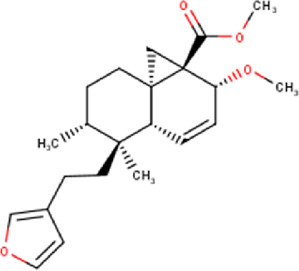	Diterpenoid	Zhang et al. (2016)
51	19.74	484.54	C_27_H_32_O_8_	Viscoflavone B 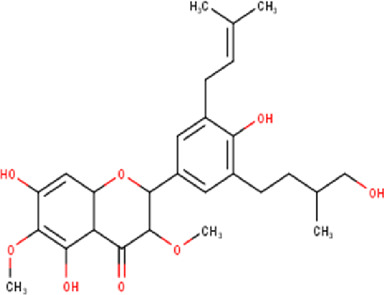	Flavonoid	—
52	21.49	332.44	C_20_H_28_O_4_	Vishautriwaic acid 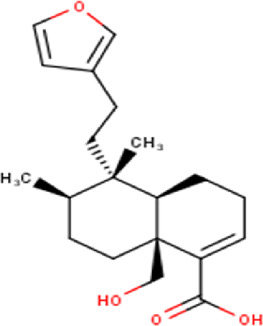	Diterpenoid	Sagara et al. (2021)
53	22.07	412.43	C_23_H_24_O_7_	Viscosol 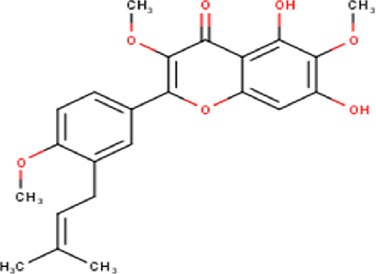	Flavonoid	Sagara et al. (2021)
54	22.25	466.53	C_27_H_30_O_7_	Viscoflavone A 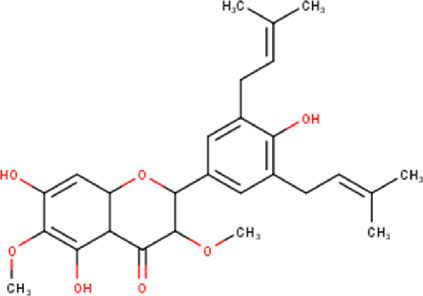	Flavonoid	—
55	24.10	782.92	C_43_H_58_O_13_	Unknown	—	—
56	24.35	255.44	C_16_H_33_NO	Palmitamide 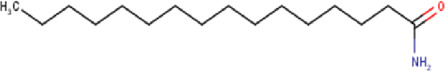	Fatty amide	—
57	24.61	281.48	C_18_H_35_NO	9-Octadecenamide 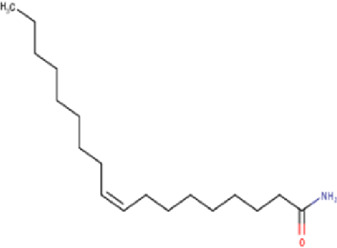	Fatty amide	—

Some chemical constituents in other reports as Isorhamnetin (Al-Snafi, 2017), kaempferol (Omosa et al., 2010), quercetin [24], aliarin (Teffo et al., 2010), methyl dodovisate B (Niu et al., 2010), hardwickiic acid (Niu et al., 2010), fraxetin (Kaigongi et al., 2020), chlorogenic acid (Beshah et al., 2020), p-coumaric acid (Beshah et al., 2020), rutin, viscosol, catechin, and dodonic acid (Kaigongi et al., 2020) were determined by chromatographic analysis and showed antibacterial activity. Otherwise, some compounds in this study have been reported in the first time such as sucrose, quinic acid, shikimic acid, adenosine, N-(1-deoxy-1-fructosyl) isoleucine, tryptophan, 6,8-C-dihexosylnoreugenin, 6,8-hexosylnaringenin, procyanidin B2, 6-(3-benzoyloxy-2-hydroxypropoxy)-3,4,5- trihydroxyoxane-2-carboxylic acid, quercetin 3-O-[rhamnosyl-(1→2)- [rhamnosyl-(1→6)]-glucoside], 3-O-Feruloylquinic acid, kaempferol-3-O-[rhamnosyl-(1→2)- [rhamnosyl-(1→6)]-galactoside], 1-O-methyl-2-acetyl-3-p-coumaryl-myo- inositol, kaempferol-3-O-rutinoside, isorhamnetin 3-O-[α-L-Rhamnopyranosyl- (1→6)-β-D-galactopyranoside, Loliolide, 3,8,16-Trihydroxy-13-labden-15,16-olide; 5,7-dihydroxy-3′,4′,5′-trimethoxyfiavone, 7-O-[β-D-glucuronopyranosyl-(1→2)-β-Dglucopyranoside], naringenin, kauralexin B3, 3′,5,5′-Trihydroxy-7-methoxy flavanone, tamarixetin, mkapwanin, viscoflavone B, palmitamide, and 9-octadecenamide.

### 3.2 Cytotoxic activity of *D. viscosa*

The cytotoxic effect of the hydroethanolic extract of *D. viscosa* and 5-FU (conventional treatment) was examined in colon cancer cells (SW480 and SW620) and nonmalignant cell lines (HaCaT and CHO-K1) using the sulforhodamine B test. As demonstrated in Table 2, the IC_50_ values of the extract were equivalent to those seen for the chemotherapeutic drug at both 24 and 48 h. Furthermore, the extract exhibited slightly better selectivity than 5-FU, as shown by a decreased toxicity towards nonmalignant cells. In addition, it was discovered that cellular morphology was substantially altered when treated with the extract, demonstrating size and shape modifications, whilst the control vehicle (DMSO) seemed normal and healthy. These morphological abnormalities increased in a time- and dose-dependent way, resulting in a significant decrease in the number of cells compared to the control, indicating either a movement toward cell death or a halt in the cell cycle.

**TABLE 2:** Cytotoxic activity of *D. viscosa* on colon cancer cell lines.

24 h								
Treatment	IC_50_ µg/mL				Selectivity index (SI)			
	SW480	SW620	HaCaT	CHO-K1	HaCaT/SW480	CHO-K1/SW480	HaCaT/SW620	CHO-K1/SW620
Control	NI	NI	NI	NI	—	—	—	—
*Dodonaea viscosa*	129.8 ± 2.78	87.7 ± 3.11	98.9 ± 4.65	80.4 ± 4.33	0.76	0.62	1.13	0.92
5-FU	200.8 ± 17.3	116.9 ± 12.0	50.8 ± 17.4	70.7 ± 5.2	0.25	0.35	0.43	0.60

48 h								
Treatment	IC_50_ µg/mL				Selectivity index (SI)			
Control	NI	NI	NI	NI	—	—	—	—
*Dodonaea viscosa*	37.0 ± 1.58	28.2 ± 1.69	51.5 ± 3.53	41.4 ± 2.32	1.39	1.12	1.83	1.47
5-FU	22.7 ± 1.3	23.5 ± 2.12	20.2 ± 1.0	22.5 ± 1.4	0.89	0.99	0.86	0.96

NI, non-inhibition; 5-FU, 5-fluorouracil; SI, values higher than 1 are considered selective for tumor cells.

Other authors have evaluated *D. viscosa* using different models. Thus, Malik et al. (2022) reported that the leaf extracts of *D. viscosa* possess toxicity against shrimp larvae with 70% mortality. Besides, they reported that this cytotoxic activity might be due to defensive secondary metabolites as flavonoids, phenols, saponins or other compounds. In contrast, Herrera-Calderon et al. (2020) reported that *D. viscosa* possess a slight cytotoxic effect against colon cancer cells (HT-29) in comparison to 5-FU, besides, they found this extract does not possess any detectable cytotoxic effect on epidermal cells from mouse. These findings highlight the importance of evaluating the extract using different models to explore the real potential of *D. viscosa.*

### 3.3 Antiproliferative activity of *D. viscosa*

**FIGURE 2:**
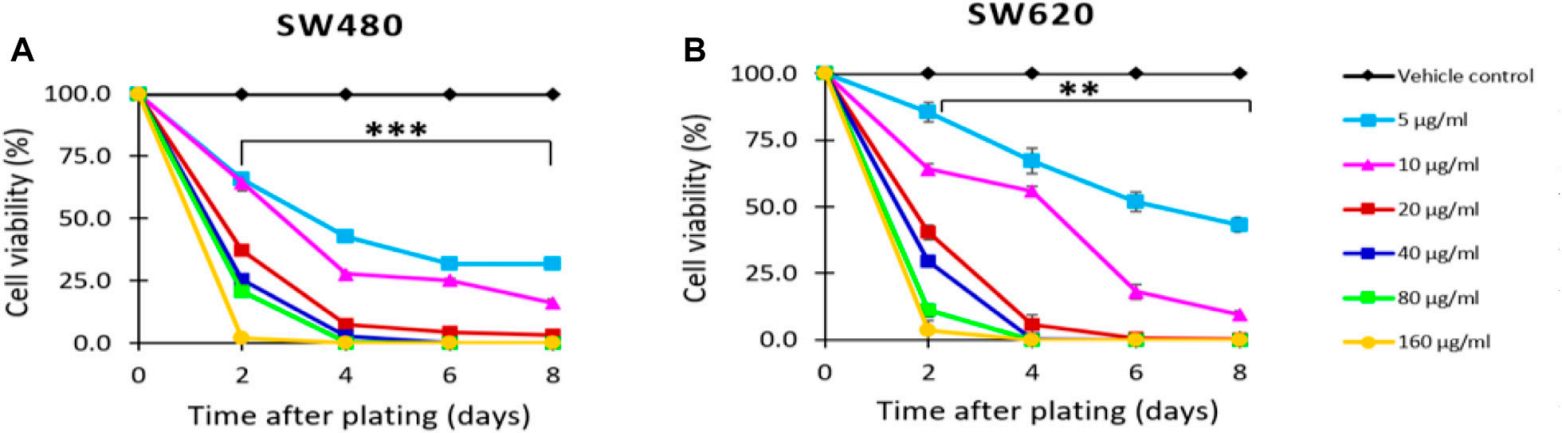
Effect of *D. viscosa* extract on the proliferation of SW480 **(A)** and SW620 **(B)** cells. Data are provided as the mean ± standard error (SE) of at least three replicates (**p < 0.01; ***p < 0.001).

The antiproliferative activity of *D. viscosa* was assessed against SW480 and SW620 colon cancer cells using the colorimetric assay sulforhodamine B. Based on the data depicted in Figure 2, it was determined that *D. viscosa* exhibits strong antiproliferative action in both cell lines beginning on day 2 at concentrations as low as 5 g/mL. In addition, when cells were treated with the extract, the assessment under an optical microscope revealed that the cells underwent morphological changes, including alterations in size and form, indicating that they were in the process of dying. Cao et al. (2009) evaluated different extracts of the same plant in a different cancer model. They reported that triterpenoid saponins of *D. viscosa* possess antiproliferative activity against the A2780 human ovarian cancer cell line (Cao et al., 2009). So, considering that chemoprevention involves the reversion, suppression, or prevention either the initial phases of carcinogenesis or the progression of premalignant cells to invasive disease using natural, synthetic, or biological agents (Steward and Brown, 2013), our findings revealed that the natural extract of *D. viscosa* could exert chemopreventive potential inducing antiproliferative activity in colorectal cancer, suggesting it could also be an important candidate for future investigations in this matter.

### 3.4 Changes in mitochondrial membrane potential (ΔΨm) and plasma membrane integrity

**FIGURE 3:**
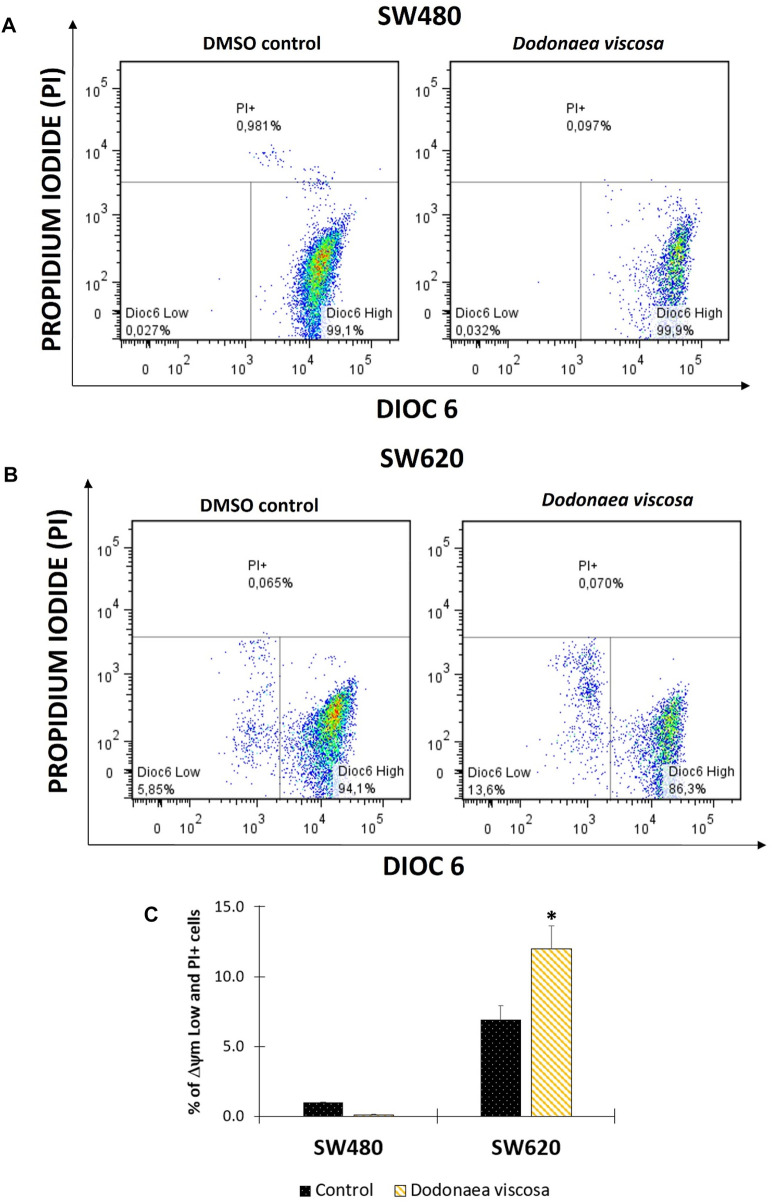
Flow cytometric analysis of cells stained with DiOC6 and PI. Mitochondrial membrane potential (ΔΨm) in SW480 **(A)** and SW620 **(B)** cells. Representation of data with total ΔΨm Low plus PI + cells in bar chart form **(C)**; Dioc6 High: live cells with high ΔΨm; Dioc6 Low: cells in latency that lose ΔΨm; PI+: Cells with membrane damage or dead cells. The used concentrations were 37.0 ± 1.58 μg/mL for SW480 and 28.2 ± 1.69 μg/mL for SW620.

Mitochondrial membrane potential (ΔΨm) plays a significant role in apoptosis (Ly et al., 2003). Changes in ΔΨm have been shown to precede caspase activation and cell death. Because of this, the hydroethanolic extract of *D. viscosa* was examined 48 h after treatment to determine alterations in mitochondrial membrane potential. Using a lipophilic dye (Dioc6(3)) that is specific for the mitochondria of living cells, flow cytometry was performed. This accumulates in the mitochondrial matrix and is discharged into the cytosol following the decrease in ΔΨm. In addition, propidium iodide was utilized concurrently to assess alterations in the integrity of the cell membrane (Herrera-R et al., 2019). According to the results shown in Figure 3, it was observed that the extract of *D. viscosa* did not alter the mitochondrial membrane potential of SW480 cells, nevertheless, it was observed a significant mitochondrial depolarization in SW620 cells. A similar study was reported by Mossa and Al-Shawi (2015) who evaluated the extract of *D. viscosa* in human breast MDA-MB231 cancer cells and observed that membrane potential decreased with higher doses of the extract (Mossa and Al-Shawi, 2015). Considering that those changes in mitochondrial membrane potential are associated with apoptosis and cell death, our findings suggest this could be a possible mechanism related to the activity of the extract of *D. viscosa* in SW620 colon cancer cells.

### 3.5 Effect of the hydroethanolic extract of *D. viscosa* on cell cycle distribution

**FIGURE 4:**
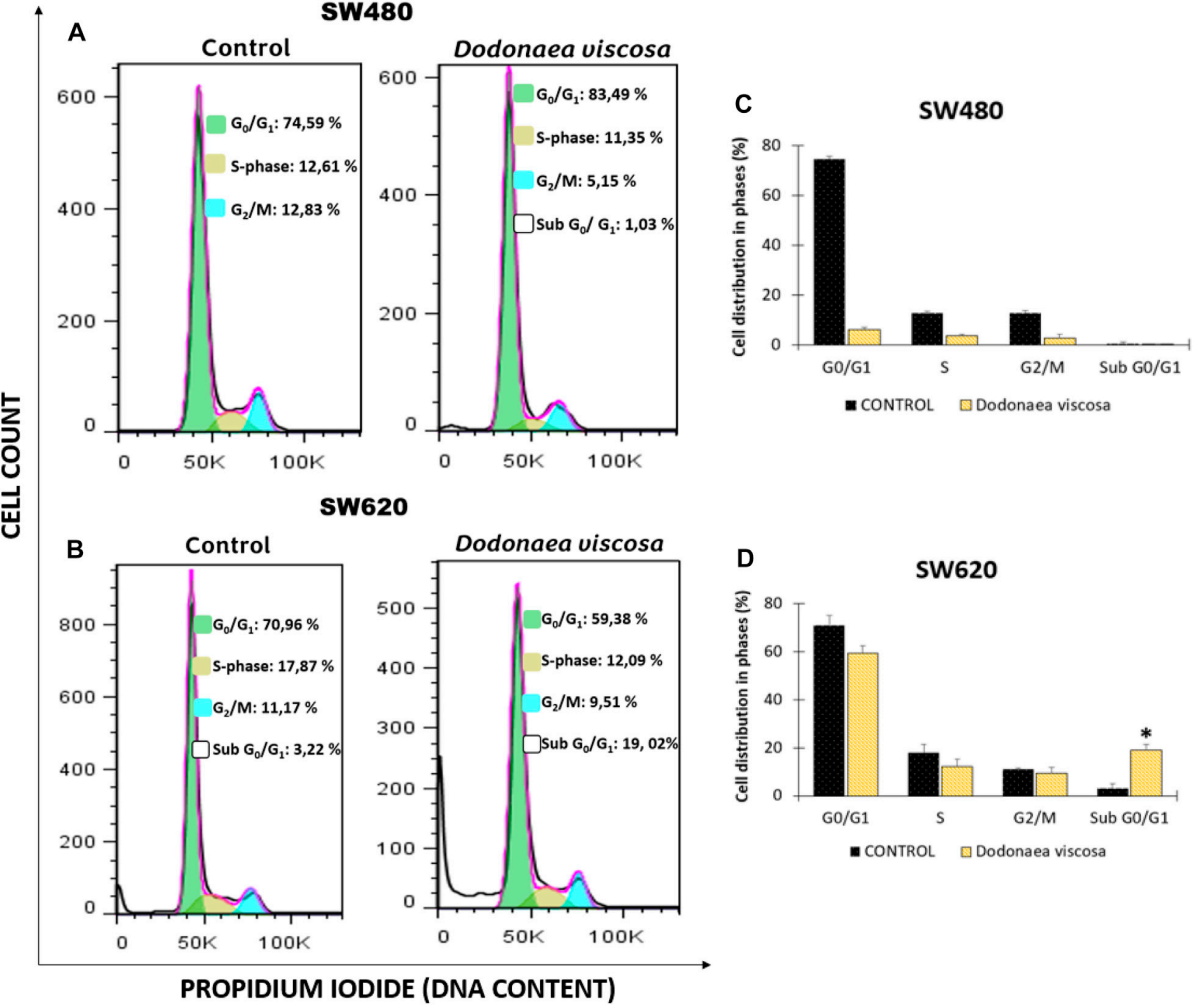
Cell cycle analysis of SW480 **(A)** and SW620 **(B)** cells following treatment with DMSO (1%), or the extract of *D. viscosa*. Data presentation using a bar chart **(C,D)**. Cells were PI-labeled and subjected to a DNA flow cytometry analysis after 48 h of treatment. The statistics show the proportion of cells in each stage of the cell cycle. Each experiment was carried out twice and had similar results. Statistical significance was defined as *p* < 0.05 (*p < 0.05). The used concentrations were 37.0 ± 1.58 μg/mL for SW480 and 28.2 ± 1.69 μg/mL for SW620.

The impact of *D. viscosa* on SW480 and SW620 cell cycle distribution was studied using propidium iodide (PI) by flow cytometry. This fluorescent dye intercalates into the main groove of double-stranded DNA to provide a highly fluorescent signal that identifies the percentage of cells in one of the three interphase phases. Cells were treated with either 1% DMSO alone or the IC_50_-determined extract (the same concentration was used in all experiments). Following 48 h of treatment, cells were stained with PI and examined using DNA flow cytometry. Figure 4 depicts an example histogram for each cell line. In SW480 cells (Figures 4A, C), there were no significant differences between the control and the cell cycle distribution. In contrast, in SW620 cells (Figures 4B, D), a large number of cells were undergoing apoptosis, as shown by a high accumulation of cells in sub-G0/G1 and a minor drop in the other phases. These results are comparable with those published by Mossa and Al-Shawi (2015), who discovered that the hydroethanolic extract of *D. viscosa* promotes cell cycle arrest at the S phase in MDA-MB231 human breast cancer cells.

On the other hand, different authors have reported that the activity of *D. viscosa* is attributed to different polyphenols detected in the plant, including apigenin, rutin, quercetin and flavonoids, among others, who have demonstrated a plethora of biological activities (Lawal and Yunusa, 2013; Malik et al., 2022). These findings agree with those reported by Cai et al. (2011) which found that the apigenin might be responsible for the observed activity in human hepatoma Huh7 cells, through arresting in the cell cycle at the G2/M phase (Cai et al., 2011). Likewise, Weiqun Wang and others (Wang et al., 2004) indicated that treatment with different analogs of apigenin (chrysin, acacetin, kaempferol, luteolin, or quercetin) resulted in the cell-cycle arrest at the G2/M phase in a dose-dependent manner in SW480 cells. Considering the urgent need for the development of new treatment strategies with the ability to target cancer cells without harming normal cells, the antiproliferative activity of *D. viscosa* observed in this study highlights the importance of evaluating natural sources as potential treatments or adjuvants even in those cases associated with chemoresistant phenotype.

### 3.6 Apoptosis induction by the hydroethanolic extract of *D. viscosa*

**FIGURE 5:**
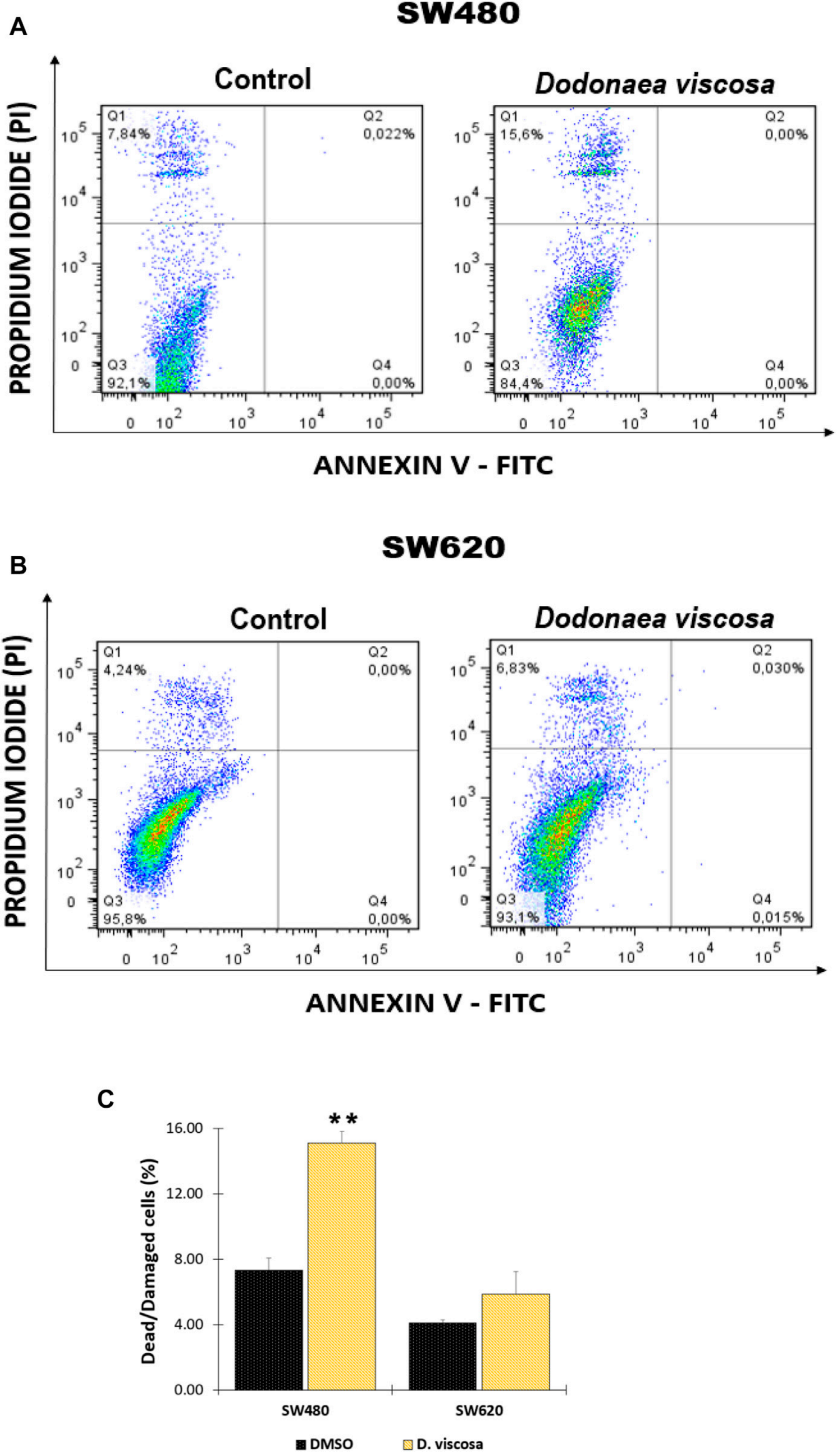
Analysis of apoptosis in SW480 **(A)** and SW620 **(B)** cells after treatment with *D. viscosa* extract. Bar chart representation of injured and dying cells that have lost their membrane integrity (Q1 + Q2) **(C)**. One of at least two different experiments is represented by the histograms. Q1 + Q2: Cells that lost membrane integrity, including late apoptotic, dead, necroptotic, secondary necrotic, and other cells; Q3: Viable cells; Q4: Early apoptotic cells. Cells used as controls received 1% DMSO treatment. All experiments were carried out twice and produced comparable outcomes. Statistical significance was determined by *p* values less than 0.05 (**p < 0.01). The used concentrations were 37.0 ± 1.58 μg/mL for SW480 and 28.2 ± 1.69 μg/mL for SW620.

The plasma membrane’s direct barrier to the extracellular environment is essential for the maintenance of tissue homeostasis. Loss of membrane integrity can be associated with apoptosis, post-apoptotic secondary necrosis, necroptosis, and other kinds of programmed cell death, which terminates cellular life. Using a double staining with annexin-FITC and propidium iodide, it was determined if the extract of *D. viscosa* produces plasma membrane damage and likely cell death, which was confirmed by prior findings for mitochondrial membrane potential and cell cycle distribution. Figure 5 depicts the observed results. After 48 h of exposure, it was discovered that the extract stimulates plasma membrane breakdown in SW480 (Figure 4A) and SW620 (Figure 5B) cells, as shown by the movement of the cells onto the top quadrant with positive propidium iodide staining. These results were statistically significant in SW480 cells (Figures 5A–C), confirming that the extract triggers cell death independent of mitochondria in SW480 cells.

According to (Mossa and Al-Shawi, 2015), increasing doses of the hydroethanolic extract of *D. viscosa* elicit apoptosis and a decrease in mitochondrial membrane potential in human breast cancer MDA-MB231 cells. In addition, it has been postulated, as in the case of cell cycle analysis, that the activity might be linked to the presence of various polyphenols (Malik et al., 2022). Cai et al. (2011) discovered that apigenin, one of the chemicals identified in the extract of *D. viscosa*, significantly enhances apoptosis in human hepatocellular carcinoma Huh7 cells. These findings suggest that *D. viscosa* could be a promising candidate for the chemoprevention of various cancers, and it is essential to continue researching this natural source to identify both the molecules responsible for its chemopreventive activity and the signaling pathway associated with the mechanism of action of these molecules.

### 3.7 Determination of apoptotic biomarkers

**FIGURE 6:**
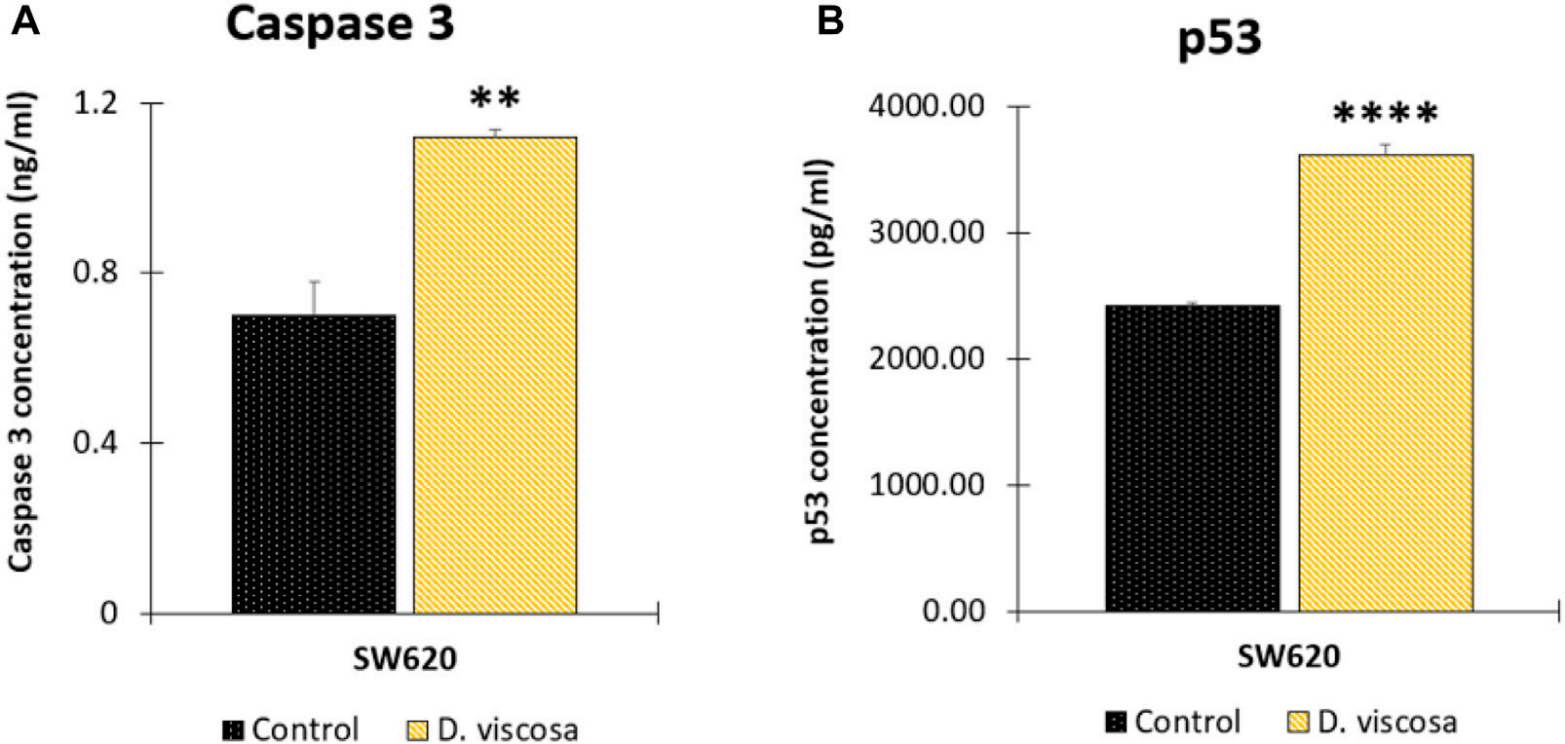
Determination of apoptotic biomarkers in SW620 cells. Cells were harvested 48 h after treatment with the extract or the vehicle control (DMSO 1%). Protein levels of caspase 3 **(A)** and tumor suppressor protein p53 **(B)**. Data are presented as the mean ± SE of two independent experiments. *p* values lower than 0.05 were considered statistically significant (**p < 0.01; ****p < 0.001). The used concentrations were 37.0 ± 1.58 μg/mL for SW480 and 28.2 ± 1.69 μg/mL for SW620.

To complement the previous findings and get closer to the possible mechanism associated with the hydroethanolic extract of *D. viscosa*, we evaluated different apoptotic biomarkers. Firstly, considering that apoptosis is a highly regulated process, we evaluated caspases 3 and −7 which are the hallmarks of the degradation phase of apoptosis and are responsible for initiating cell shrinkage, membrane blebbing, and DNA fragmentation. According to the results, it was found that the hydroethanolic extract was only active on SW620 cells (Figure 6A), causing an important increase in the concentration of the active form of this protease, suggesting that one of the possible mechanisms of the extract on SW620 cells could be related to apoptosis mediated by caspase 3. On the contrary, in SW480 cells, it was not observed an important change caused by the extract (data not shown). We also evaluated caspase 8 to understand if the extract could be responsible for death receptor-induced cell death, however, we did not observe any changes on this protease (data not shown) which suggests that the apoptotic process in SW620 cells is mediated by mitochondria, confirming our previous findings on mitochondrial membrane potential (ΔΨm). In addition, considering the pivotal role of the tumor-suppressor protein p53 in the regulation of different cellular processes such as apoptosis (Xie et al., 2001; Mantovani et al., 2019), we evaluated if the extract of *D. viscosa* could modulate the expression of this protein. According to the results, this extract caused an important increased in the concentration levels of this protein in SW620 cells (Figure 6B) but not in SW480 cells (data not shown). This finding is very important because the tumor suppressor protein p53 is mutated in SW620 cells, and our experimental results suggest that the extract could activate the tumor suppressor protein in SW620 cells. These results are supported by those investigations reported by other authors who have demonstrated that p53 retains some of the functions and maintains residual DNA-binding ability (Bykov et al., 2014), being possible to activate it both *in vitro* and *in vivo* through different mechanisms (Selivanova and Wiman, 2007; Bykov et al., 2014), complementing our previous findings and suggesting that the apoptotic process induced by this extract could be mediated by the intrinsic pathway in response to the activation of p53. All our findings together with the previous investigations in the same matter suggest that *D. viscosa* could potentially be considered as chemopreventive agent or adjuvant in the treatment of colorectal cancer, even in those cases with resistance to conventional chemotherapy because of the lack of p53 expression or function, and thus, it is necessary to carry out further investigations with this natural source to discover new molecules against this disease.

Although in this study, it was used the hydroalcoholic extract, some compounds found in the chromatographic analysis, specially flavonoids and their derivates might be responsible for the anticancer activity like quercetin through downregulation, this flavonoid primarily targets the pro-survival Bcl-2 component of the p53 pathway, the members of the PI3K/AKT/mTOR, Wnt/-catenin, NF-B, and MAPK signaling pathways, the MMPs, the anabolism with AMPK as a marker, and the stress response to reactive oxygen species (ROS) (Neamtu et al., 2022). Other flavonoids like rutin can also trigger apoptosis in cancer cells by activating p53. In colon cancer cells like HCT cell line, rutin treatment activated caspase-3. In addition, it has been demonstrated that rutin activates both the intrinsic and extrinsic apoptotic pathways in colon cancer (HT-29) cells by upregulating caspases. This evidence significantly supports rutin’s ability to induce apoptosis by activating both the intrinsic (mitochondria-mediated) and extrinsic (death receptor-mediated) apoptotic pathways (Pandey et al., 2021).

## 4 Conclusion

The hydroethanolic extract of *D. viscosa* exhibited cytotoxic and antiproliferative activity on human colon cancer cell lines SW480 and SW620. We hypothesized that the probable mechanism in the metastatic derivative SW620 could be associated with an intrinsic apoptosis via regulation of caspase 3 and the tumor suppressor protein p53, while in SW480 the mechanism seems not to be involving a mitochondrial process. This is an approach toward the possible role of this plant in colorectal cancer, however, further studies are needed to explore the full potential of *D. viscosa* as chemopreventive agent in the treatment of colorectal cancer.

## Data Availability

The original contributions presented in the study are included in the article/Supplementary Material, further inquiries can be directed to the corresponding authors.
