# Supplementary material for: Dodonaea viscosa Jacq. induces cytotoxicity, antiproliferative activity, and cell death in colorectal cancer cells via regulation of caspase 3 and p53
Source: Front Pharmacol. 2023 Jun 23;14:1197569. doi: 10.3389/fphar.2023.1197569 (PMC10326442; doi:10.3389/fphar.2023.1197569)
Supplement: Supplementary file 1 [file Table1.DOCX]

**Supplementary material**

Table S1: List of Phytochemical compounds found in the hydroalcoholic extract of *Dodonaea viscosa* by UPLC-MS.

| N° | Retention time | MS-ES^-^ | MS^2^ | Error  ppm | MS-ES^+^ | MS^2^ | Error  ppm | Molecular Formula/  Molecular Mass | Proposed  compound | Chemical group |
| --- | --- | --- | --- | --- | --- | --- | --- | --- | --- | --- |
| 1 | 1.68 | 341.1089 | 179.0554 119.0340 101.0234 89.0233 71.0127 59.0127 | 2.99 | - | - |  | C_12_H_22_O_11_  342.30 | Sucrose | Glycosyl glycoside |
| 2 | 1.79 | 191.0556 | 173.0449 127.0392 93.0335 85.0284 | 2.86 | - | - |  | C_7_H_12_O_6_  192.17 | Quinic acid | Organic acid |
| 3 | 1.86 | 173.0449 | 155.0342 137.0235 129.0184  111.0441 93.0335 73.0284 | 2.66 | - | - |  | C_7_H_10_O_5_  174.15 | Shikimic acid | Organic acid |
| 4 | 2.39 | - | - |  | 268.1039 | 136.0618 115.0392 85.0288 | -0.56 | C_10_H_13_N_5_O_4_  267.24 | Adenosine | Ribonucleoside |
| 5 | 2.39 | - | - |  | 276.1440 [M^+^H-H_2_O] | 258.1334 230.1385 212.1279 161.0681 132.1019 86.0969 | -0.56 | C_12_H_23_NO_7_  293.31 | N-(1-deoxy-1-fructosyl) isoleucine | Aminoacid |
| 6 | 5.45 | - | - |  | 188.0706 [M^+^H-NH_3_] | 170.0599 146.0600 144.0808 118.0653 | -0.03 | C_11_H_12_N_2_O_2_  204.22 | Tryptophan | Aminoacid |
| 7 | 7.54 | - | - |  | 355.1023 | 337.0916 319.0811 301.0705 289.0704 273.0756 259.0599 235.0600 205.0494 | -0.08 | C_16_H_18_O_9_  354.31 | 6,8-C-dihexosylnoreugenin | Chromones |
| 8 | 8.75 | 353.0875 | 191.0553 179.0342 161.0236 135.0442 |  | 355.1021 | 285.0101 163.0388 91.0578 | -0.62 | C_16_H_18_O_9_  354.31 | Chlorogenic acid | Phenolic acid |
| 9 | 9.03 | 456.30001 | 475.1526 415.1034 385.0924 355.0819 313.0715 | 1.89 | - | - |  | C_27_H_32_O_5_  456.34 | 6,8-hexosylnaringenin | Flavonoid glycosides |
| 10 | 9.52 | 577.1531 | 407.0764 339.0869  289.0714 245.0815 203.0707 161.0235 125.0234 | 1.87 | - | - |  | C_30_H_26_O_12_  578.529 | Procyanidin B2 | Catechin |
| 11 | 9.95 | 289.0715 | 245.0815 203.0706 179.0342 151.0391 125.0234 109.0284 | 2.99 | 291.0862 | 207.0650 179.0700 165.0545 147.0439 139.0389 123.0442 | -0.57 | C_15_H_14_O_6_  290.27 | Catechin | Catechin |
| 12 | 10.23 | 371.0891 | 249.0611 121.0285 113.0233 99.0077 85.0284 75.0076 | 2.26 | - | - |  | C_16_H_20_O_10_  372.32 | 6-(3-benzoyloxy-2-hydroxypropoxy)-3,4,5- trihydroxyoxane-2-carboxylic acid | Organic acid |
| 13 | 10.27 | 337.0927 | 191.0553 173.0447  163.0392 93.0334 | 2.54 | - | - |  | C_16_H_18_O_8_  338.31 | p-Coumaroylquinic acid | Organic acid |
| 14 | 10.35 | 755.2038 | 489.1028 300.0272 271.0244 255.0292 178.9978 151.0026 | 1.17 | - | - |  | C_33_H_40_O_20_  756.66 | Quercetin 3-O-[rhamnosyl-(1→2)- [rhamnosyl-(1→6)]-glucoside] | Flavonoid glycosides |
| 15 | 10.40 | 863.1831 | 411.0717 289.0714 245.0816 161.0263 125.0234 | 1.53 | 865.1971 | 543.0907 409.0912 287.0547 257.0441 247.0598 139.0388 | -0.42 | C_45_H_36_O_18_  864 | Unknown (procianidin trimer) | - |
| 16 | 10.64 | 367.1032 | 191.0553 173.0447 134.0363 111.0441  93.0334 | 2.32 | - | - |  | C_17_H_20_O_9_  368.34 | 3-O-Feruloylquinic acid | Organic acid |
| 17 | 10.72 | 739.2072 | 575.1411 284.0323 255.0295 178.9975 151.0024 | 1.66 | - | - |  | C_33_H_40_O_19_  740.66 | Kaempferol-3-O-[rhamnosyl-(1→2)- [rhamnosyl-(1→6)]-galactoside] | Flavonoid glycosides |
| 18 | 10.85 | 381.1188 | 339.1031 321.0974 163.0392 145.0285 119.0492 | 1.99 | - | - |  | C_18_H_22_O_9_  382.36 | 1-O-methyl-2-acetyl-3-*p*-coumaryl-myo- inositol | Organic acid |
| 19 | 11.08 | 609.1460 | 343.0456 300.0272 271.0244 178.9978 151.0027 | 1.62 | 611.1605 | 345.0605 303.0498 129.0547 85.0290 71.0498 | -0.23 | C_27_H_30_O_16_  610.53 | Rutin | Flavonoid glycosides |
| 20 | 11.42 | 593.1512 | 447.0902 327.0506 284.0323 255.0294 151.0028 | 1.95 | 595.1658 | 426.8344 329.0651 287.0548 174.9431 85.0289 | 0.04 | C_27_H_30_O_15_  594.52 | kaempferol-3-O-rutinoside | Flavonoid glycosides |
| 21 | 11.63 | 623.1614 | 357.0610 314.0429 299.0193 271.0244 243.0294 151.0027 | 1.25 | 625.1761 | 359.0750 317.0655 302.0418 129.0547 85.0290 71.0498 | -0.35 | C_28_H_32_O_16_  624.55 | Isorhamnetin 3-O-[α-L-Rhamnopyranosyl- (1→6)-β-D-galactopyranoside | Flavonoid glycosides |
| 22 | 12.21 | - | - |  | 197.1171 | 179.1066 161.0960 151.1117  135.1169 133.1012 121.0649 107.0858 | -0.56 | C_11_H_18_O_3_  198.26 | Loliolide | Benzofuran |
| 23 | 12.90 | 465.2125 | 303.1602 285.1492 259.1699 113.0233 101.0233 89.0232 71.0127 | 1.18 | 484.2541 [M^+^NH_4_] | 287.0164 249.1119 241.1586 231.1015 217.0858 173.0961 127.0390 85.0289 | -0.10 | C_24_H_34_O_9_  464.52 | Unknown | - |
| 24 | 13.11 | 287.0559 | 259.0608 243.0658 215.0707 201.0549 178.9978 151.0027 125.0234 | 3.02 | - | - |  | C_15_H_12_O_6_  288.25 | 4',5,7-Trihydroxydihydroflavonol | Flavonoid |
| 25 | 13.40 | 351.2774 | 315.1961 289.2169 271.2064 259.2063 189.1278 133.1013 | 0.91 | - | - |  | C_20_H_32_O_5_  352.47 | 3,8,16-Trihydroxy-13-labden-15,16-olide | Diterpenoid |
| 26 | 13.45 | - | - |  | 653.2075 | 507.1464 345.0967 330.0729 312.0624 284.0440 | 0.03 | C_30_H_36_O_16_  652.60 | 5, 7-dihydroxy-3' ,4' ,5'-trimethoxyfiavone  7-O-[β-D-glucuronopyranosyl-(1→2)-β-Dglucopyranoside] | Flavonoid glycosides |
| 27 | 14.00 | 480.2332 | 383.2436 319.1911 291.1962 277.1805 263.2012 189.1277 | 1.49 | - | - |  | C_22_H_36_O_5_  380.52 | 15,16-Epoxy-6,13,14,15,16-pentahydroxy-3- cleroden-18-oic acid, 15,16-dimethyl ether (isomer 1) | Diterpenoid |
| 28 | 14.24 | 380.2333 | 383.2436 319.1911 291.1961 277.1805 261.1856 189.1277 | 1.56 | - | - |  | C_22_H_36_O_5_  380.52 | 15,16-Epoxy-6,13,14,15,16-pentahydroxy-3- cleroden-18-oic acid, 15,16-dimethyl ether (isomer 2) | Diterpenoid |
| 29 | 14.50 | 363.1810 | 319.1911 275.2013 259.1699 217.1228 189.1277 173.0963 | 2.08 | - | - |  | C_20_H_28_O_6_  364.43 | 6,12-dioxo-7-labdene-15,18-dioic acid (isomer 1) | Diterpenoid |
| 30 | 15.11 | 271.0611 | 177.0185 169.0134 151.0027 119.0492 107.0127 93.0334 | 3.58 | 273.0757 | 171.0287 153.0182 147.0440 123.0441 119.0493 | -0.04 | C_15_H_12_O_5_  272.25 | Naringenin | Flavonoid |
| 31 | 15.33 | - | - |  | 299.2007 [M+H-H_2_O] | 281.1897 253.1949 211.1479 197.1324 173.1324 157.1012 145.1012 131.0856 119.0857 | 0.49 | C_20_H_28_O_3_  316.44 | Kauralexin B3 | Diterpenoid |
| 32 | 15.45 | 395.2072 | 377.1968 345.1705 333.2070 273.1857  235.3335 189.1278 110.0363 | 1.89 | - | - |  | C_21_H_32_O_7_  396.48 | Unknown 1 (isomer 1) | - |
| 33 | 15.71 | 395.2071 | 377.1967 345.1704 333.2068 273.1857 235.3335 189.1277 110.0363 | 1.75 | - | - |  | C_21_H_32_O_7_  396.48 | Unknown 1  (isomer 2) | - |
| 34 | 15.81 | 329.0665 | 314.0430 299.0194 271.0245 | 2.71 | 331.0810 | 316.0573 298.0469 281.0441 270.0518 | -0.72 | C_17_H_14_O_7_  330.29 | 4',5,7-Trihydroxy-3,6-dimethoxyflavone | Flavonoid |
| 35 | 15.81 | 347.1861 | 303.1962 285.1856 273.1858 259.2064 189.1278  135.0806 85.0284 | 0.98 | - | - |  | C_20_H_28_O_5_  348.43 | 6-Hydroxy-3,13-clerodadien-16,15-olid-18- oic acid | Diterpenoid |
| 36 | 15.91 | 363.1811 | 319.1911 273.1857 259.1700 231.1749 217.1228 189.1278 173.0963 | 2.49 | - | - |  | C_20_H_28_O_6_  364.439 | 6,12-dioxo-7-labdene-15,18-dioic acid (isomer 2) | Diterpenoid |
| 37 | 16.13 | 301.0715 | 165.0185 135.0442 109.0285 | 2.87 | 303.0863 | 185.0443 167.0338 163.0389 145.0283 | -0.05 | C_16_H_14_O_6_  302.28 | 3',5,5'-Trihydroxy-7-methoxyflavanone | Flavonoid |
| 38 | 16.25 | 415.1396 | 400.1161 385.0927  357.0977 | 0.72 | - | - |  | C_22_H_24_O_8_  416.42 | Aliarin | Flavonoid |
| 39 | 16.28 | 363.1812 | 319.1911 275.2013 245.1906 217.1229 189.1277 173.0963 | 2.57 | - | - |  | C_20_H_28_O_6_  364.43 | 6,12-dioxo-7-labdene-15,18-dioic acid (isomer 3) | Diterpenoid |
| 40 | 16.41 | - | - |  | 317.1020 | 197.0443 182.0207 147.0439 | -0.48 | C_16_H_12_O_7_  316.26 | Tamarixetin | Flavonoid |
| 41 | 16.68 | 345.1707 | 301.1808 273.1859 243.1751 215.1073 169.1016 83.0491 | 1.31 | - | - |  | C_20_H_26_O_5_  346.42 | Unknown 2  (isomer 1) | - |
| 42 | 16.88 | 345.1705 | 301.1807 273.1859 243.1751 219.1385 169.1015 83.0491 | 0.96 | - | - |  | C_20_H_26_O_5_  346.42 | Unknown 2  (isomer 2) | - |
| 43 | 17.19 | 343.0823 | 328.0588 313.0354 298.0170 285.0403 270.0167 | 3.06 | 345.0968 | 330.0728 312.0625 287.0546 269.0441 121.0285 | -0.14 | C_18_H_16_O_7_  344.32 | Penduletin | Flavonoid |
| 44 | 17.38 | 285.0766 | 165.0185 119.0492 93.0335 | 3.09 | 287.0912 | 167.0338 147.0439 119.0493 | -0.73 | C_16_H_14_O_5_  286.28 | Sakuratenin | Flavonoid |
| 45 | 18.11 | 313.0717 | 298.0480 283.0246 269.0453 255.0296 | 3.37 | - | - |  | C_17_H_14_O_6_  314.29 | 3,5-dihydroxy-4,7-dimethoxyﬂavone | Flavonoid |
| 46 | 18.39 | 343.0822 | 328.0588 313.0354 285.0403 270.0168 178.9979 | 2.77 | 345.0966 | 330.0729 312.0624 287.0546 281.0441 135.0439 | -0.88 | C_18_H_16_O_7_  344.32 | Santin | Flavonoid |
| 47 | 18.79 | 329.1756 | 299.1651 255.1752 161.0660 | 1.14 | - | - |  | C_20_H_26_O_4_  330.42 | Mkapwanin | Diterpenoid |
| 48 | 18.96 | 331.1913 | 287.2016 189.1279 69.0335 | 1.14 | - | - |  | C_20_H_28_O_4_  332.44 | Dodonic acid | Diterpenoid |
| 49 | 19.33 | 397.1291 | 382.1057  367.0822 339.0870 270.0168 | 0.84 | - | - |  | C_22_H_22_O_7_  398.41 | Dodoviscin J | Flavonoid |
| 50 | 19.57 | 357.1707 | 313.1808 281.1545 253.1595 227.1436 217.1229 173.1328 | 1.35 | - | - |  | C_22_H_30_O_4_  358.47 | Dodovisnoid E | Diterpenoid |
| 51 | 19.74 | 483.2024 | 468.1789 453.1555 425.1621 356.0899 178.9981 | 1.03 | - | - |  | C_27_H_32_O_8_  484.54 | Viscoflavone B | Flavonoid |
| 52 | 21.49 | - | - |  | 315.1954 [M+H-H_2_O] | 297.1848 279.1742 251.1794 241.1222  149.0960 135.0805 107.0858 95.0496 81.0340 | -1.97 | C_20_H_28_O_4_  332.44 | Vishautriwaic acid | Diterpenoid |
| 53 | 22.07 | 411.1446 | 396.1213 381.0980 366.0742 353.1030 338.0796 178.9979 | 0.59 | - | - |  | C_23_H_24_O_7_  412.43 | Viscosol | Flavonoid |
| 54 | 22.25 | 465.1918 | 465.1915 450.1682 435.1447 407.1531 338.0794 | 1.08 | - | - |  | C_27_H_30_O_7_  466.53 | Viscoflavone A | Flavonoid |
| 55 | 24.10 | 781.3810 | 722.3669  397.1289  382.1055  367.0822 339.0873 297.2433 | 1.36 | 783.3944 | 679.3934 417.1538 399.1435  343.0809 315.0856 | -1.48 | C_43_H_58_O_13_  782.92 | Unknown | - |
| 56 | 24.35 | - | - |  | 256.2633 | 158.1538 130.1226 116.1071 102.0916 88.0761 74.0606 | -0.82 | C_16_H_33_NO  255.44 | Palmitamide | Fatty amide |
| 57 | 24.61 | - | - |  | 282.2789 | 265.2521 247.2419 177.1636 149.1324 135.1168 121.1013 97.1015 83.0860 69.0705 | -0.86 | C_18_H_35_NO  281.48 | 9-Octadecenamide | Fatty amide |
